# Communication Architecture in Mixed-Reality Simulations of Unmanned Systems

**DOI:** 10.3390/s18030853

**Published:** 2018-03-14

**Authors:** Martin Selecký, Jan Faigl, Milan Rollo

**Affiliations:** Faculty of Electrical Engineering, Czech Technical University in Prague, Technická 2, 166 27 Prague, Czech Republic; faiglj@fel.cvut.cz (J.F.); rollom@fel.cvut.cz (M.R.)

**Keywords:** communication architecture, mixed-reality simulations, unmanned systems, middleware, testbeds

## Abstract

Verification of the correct functionality of multi-vehicle systems in high-fidelity scenarios is required before any deployment of such a complex system, e.g., in missions of remote sensing or in mobile sensor networks. Mixed-reality simulations where both virtual and physical entities can coexist and interact have been shown to be beneficial for development, testing, and verification of such systems. This paper deals with the problems of designing a certain communication subsystem for such highly desirable realistic simulations. Requirements of this communication subsystem, including proper addressing, transparent routing, visibility modeling, or message management, are specified prior to designing an appropriate solution. Then, a suitable architecture of this communication subsystem is proposed together with solutions to the challenges that arise when simultaneous virtual and physical message transmissions occur. The proposed architecture can be utilized as a high-fidelity network simulator for vehicular systems with implicit mobility models that are given by real trajectories of the vehicles. The architecture has been utilized within multiple projects dealing with the development and practical deployment of multi-UAV systems, which support the architecture’s viability and advantages. The provided experimental results show the achieved similarity of the communication characteristics of the fully deployed hardware setup to the setup utilizing the proposed mixed-reality architecture.

## 1. Introduction

In recent years, there is a growing utilization of autonomous vehicles in areas of remote sensing and mobile sensor networks, especially if these vehicles can be operated beyond-line-of-sight (BLOS) as autonomous teams. However, development and validation of such multi-vehicle systems are complicated tasks since there are many complex issues that need to be taken into account. Some of them, such as complex interactions among individual entities (such as collision and obstacle avoidance, team cooperation, and coordinated movement), can be modeled with the help of software simulations [1,2]. However, since the goal of the development process is to deploy algorithms in the real world, software simulations and computational models by themselves are not satisfactory. The effects of real-world phenomena such as those of the weather, communication issues, sensor and actuator errors, or limited computational resources, which are difficult to model on high fidelity levels, need to be verified on real hardware assets. The proper design of a communication subsystem (as any of the vehicle-to-vehicle or vehicle-to-base options) is one of the most important challenges of multi-vehicle system development. It is crucial for the control, cooperation, and collaboration of the vehicles themselves, for the purposes of routing and topology management [3,4], message relay [5], communication constrained exploration [6], network-aware mission planning [7], or improvement in communication throughput and delay [8,9].

Experiments performed in a virtual world and a real world both have their limits and justifications, whereas simulations are much easier to set up and repeat; they can be used to model the basic functionality of the developed system and allow quick observations of results of interactions among entities and the environment. They can also save costs in cases of malfunctions and accidents. However, it is challenging for software simulations to incorporate all sources of inputs from the real world for realistic modeling of the behavior of the developed system. That is why real-world experiments should be an obligatory step in obtaining realistic results in later stages of the development of robotic systems and in the validation of the robustness of software before it is deployed.

Mixed-reality (MR) [10,11] simulations gain benefits of both these worlds. They present a world where both virtual (SW) and physical (HW) objects and entities (further referred as the SWEs and HWEs for the virtual entities and entities embodied in the physical world, respectively) can co-exist and interact in real time. A combination of entities allows the system developers to obtain more insight into the behavior of the entities (e.g., by visualization of their inner states) and to perform much cheaper and safer experiments, with a part of the system being real and another part virtualized [12,13]. MR simulations also relieve simulators from recreating entire worlds since simulation occurs in a partially real world where certain phenomena, such as noise and complex physics, do not have to be modeled [14,15].

**Figure 1:**
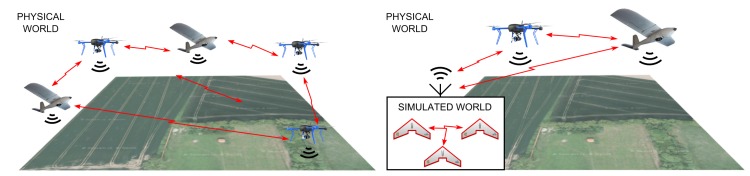
Visualization of the problem of maintaining high fidelity in communication system models in mixed-reality (MR) simulations.

One of the biggest challenges of integrating SWEs and HWEs in an MR simulation is to handle differences in the message transfer among the entities while preserving a high fidelity of the simulations (see Figure 1). The focus of this work is on designing communication architecture that would serve as a communication middleware for MR simulations and allow broadcast and directed message exchange between SWEs and HWEs while providing simultaneous wireless network simulation and movement of the entities in real time. The communication architecture must provide common addressing and message routing processes between SWEs and HWEs, as well as suitable models of media utilization and communication reachability for the MR world. In this paper, all of these problems are addressed within the context of an unmanned aerial system (UAS) as a reference system and practical deployment; however, the presented approach can be applied to other vehicular systems with direct communication among vehicles (vehicle-to-vehicle, V2V).

The communication architecture presented in this work has been utilized and verified during the development of different aviation applications within the projects funded by the Czech Ministry of Defence, the U.S. Air Force Research Lab., and the U.S. Army CERDEC [15,16,17]. The architecture has been used as a part of an MR simulation framework for the incremental development of complex systems and their verification in high fidelity testing.

The rest of this paper is organized as follows. After a discussion of related work, mechanisms of message exchange in MR systems are described in Section 3. Based on them, requirements for MR communication architecture are stated in Section 4. Then, in Section 5, a communication architecture for the MR simulations is proposed. Aspects of the fidelity of this architecture together with the precise communication modeling are addressed in Section 6. In Section 7, a set of experiments are presented to show a similarity in characteristics of the fully deployed hardware communication and the communication of entities in the MR simulation using the proposed architecture and models. Section 9 concludes the paper.

## 2. Related Work

Wireless communication properties have been intensively studied, either on the level of signal propagation, throughput, and delays or on the level of network topology control and its effect on the asset connectivity [18]. Mahdi et al. [19] conducted detailed experimental analysis, which showed that wireless connectivity among small unmanned aerial vehicles (UAVs) is challenged by the mobility and heterogeneity of the nodes, lightweight antenna design, body blockage, constrained embedded resources, and limited battery power. Pinto et al. [20] provided a model for the quality of the UAV-to-UAV link regarding packet delivery ratio as a function of distance, packet size, and orientation, which was further supported by an extensive measurement campaign. Teng et al. [21] modeled the contributors to path loss and formed a combined propagation model that more accurately reflects the reality of tested scenarios. Various types of MAC protocols, whether they are contention-based, contention-free [22], or hybrid MAC protocols [23] have been suggested for vehicle ad-hoc networks (VANETs) and tested in simulations.

Network simulators such as NS-2 [24] and OMNET++ [25] have been enhanced by supporting both wireless interactions and mobility for simulated nodes. Although these simple mobility protocols work well for simple mobile ad-hoc networks (MANETs), they fail to handle the mobility requirements of both flying ad-hoc networks (FANETs) [26] and VANETs. Use of a standard synthetic mobility model can result in incorrect conclusions, as the movement pattern can impact the networking performance of the system. FANETs require complex mobility models that involve autopilot behavior, effects of the wind, formation flights, and different mission scenarios [27]. Complex mobility models have been proposed based on an analysis of real movement traces [28]. For VANETS, there are traffic simulators such as SUMO [29] or VanetMobiSim [30] that are capable of simulating complex traffic scenarios and sophisticated driver behaviors. Specifically for the UAS reconnaissance, a pheromone-guided mobility model has been suggested in [31]. For different FANET application scenarios, various mobility models have been proposed in [32]. Despite these results, the existing approaches still fail to simulate complex operator interactions, effects of the environment, or multi-vehicle team coordination. Furthermore, the existing simulators can produce unrealistic scenarios and synthetic output [28].

Recent approaches to designing realistic simulation tools in the area of vehicular networks are presented in a survey by Silva et al. [33]. Real testbeds can solve some of the simulation’s drawbacks and can be used to test applications in native environments [34,35]. However, the costs of experiments with real testbeds are high, and they suffer from multiple issues such as scalability, limitations on specific scenarios, long preparation and running times, a lack of large-scale evaluation, and the difficulty of repetition, profiling, and modification of an experiment. Therefore, a solution is needed to overcome these issues.

The problems of real testbeds can be dealt with using an emulation concept where parts of the hardware components are emulated. The emulation data (e.g., the output of a simulator, real traces, and an event-based generator’s output ) can be then processed by the network simulator. Even though representative approaches such as NS-2 and NS-3 [36] provide emulation extensions that enable the use of simulated nodes as if they were real network nodes, this emulation method can lead to unrealistic results due to the simulators’ overhead caused by the required processing of all needed information [37]. This challenge is addressed by Ahmed et al. [38], who introduced a flexible VANET testbed architecture for VANET applications that tries to minimize the emulator overhead. However, since these approaches separate the mobility and network traffic simulation stages, they do not support a study of the interaction between these two stages, e.g., how the delays in communication influence the movement of the vehicles. Schünemann et al. [39] designed a testbed architecture that couples both traffic and network simulators and allows for the interaction between them in real time. In their testbed, vehicles broadcast information beacons (short messages with own position and other relevant data) using the network simulator. However, the architecture does not allow directed unicast messaging between the entities.

MR simulations, unlike the previously mentioned approaches, allow for simultaneous movement of entities and studying their networking capabilities on various levels of fidelity. In these simulations, parts of the hardware components as well as some entities can be simulated depending on the resources available and the required level of the fidelity. The first notion of the mixed-reality concept was presented in work by Milgram et al. [10], where the authors mainly focus on the visualization displays and overlays, e.g., for use in robot teleoperation. One of the first notions of the MR simulations used in the development of mobile robots is presented by Chen et al. in [11]. The authors used the MR simulation tool based on the 3D robot simulator Gazebo in a single robot scenario to demonstrate advantages of the combination of real entity and simulated objects for the development of the robotic system.

The most recent approach to deal with issues in the development of complex robotic systems was proposed by Jakob et al. in [14]. The authors came up with the concept of the incremental multi-level development of complex systems by the use of MR test-beds. Their concept was further extended by Selecky et al. [15] and used for the practical development of various types of multi-vehicle systems. Using the methodology of incremental development, MR simulations allow relatively simple transition between individual levels of system virtualization—from the utilization of only SWEs and emulation functionality to the utilization of a combination of HWEs and SWEs in field experiments. In the latter case, the system partially loses the advantages of pure emulation, such as the simplicity and repeatability of experiments; however, the simulation fidelity increases since information from the real world is used.

MR simulations require both broadcast and directed message exchange between SWEs and HWEs while allowing for the movement of entities together with the wireless network simulation to preserve the communication characteristics in the virtual world. Although some of the mentioned work partially solves the problem, a communication architecture that would address all required aspects, to the best of our knowledge, has not yet been described. However, since some MR simulation issues arise from a combination of two different networks (i.e., the networks of SWEs and of HWEs), some solutions can be found in the literature considering multi-network systems [40]. These issues are e.g., problems with *network partitions* (where two or more UAVs are out of the communication range of the others and/or the simulation platform, usually the ground control station (GCS)) and problems with *sharing a single address* (when HWEs need to communicate with SWEs using a single address of the GCS). Furthermore, the entities in the architecture require information about the addresses of their neighbors. This problem is addressed by the IPv6 Neighbor Discovery protocol by sending advertisements, as described in [41], by broadcasting messages to create a proximity map of the vehicles’ neighborhood [42,43], or, if the available bandwidth is an issue, by using beacons on separate communication channels [44].

Concerning the existing work and the current lack of a proper communication architecture for the MR simulation employed in the development of UASs (or any other complex vehicular system), we propose a novel communication system architecture in this paper. The proposed architecture allows for interaction and message exchange among HWEs and SWEs as well as an appropriate network simulation. The proposed solution enables high-fidelity testing in MR simulations that support a practical deployment of UASs. The proposed architecture and the related issues with the proposed solutions are described in the following sections.

## 3. Message Exchange in Mixed-Reality Simulation

In MR simulations where all entities can communicate with each other (V2V) and with GCS (similar to V2I), there are principally two networks—one among virtual entities and one among real entities (SWE and HWE) and GCS, where a simulation platform serves as a message relay between the two networks. Three types of connections are possible (SWE–SWE, HWE–HWE, HWE–SWE), and each of them employs different tools and methods for the message exchange and different processing of communication visibility of neighbors, see Table 1.

An HWE–HWE connection uses real communication devices, such as radio-frequency (RF) modems, to exchange messages directly, and the visibility of neighbors is given by a physical RF signal propagation. In the respective SWE–SWE connection, messages are exchanged directly among virtual entities using a TCP/UDP socket connection, and their mutual communication visibility needs to be modeled. The HWE–SWE connection enables the MR interaction among entities that exist in the physical world and those that are being modeled. For this type of connection, both RF modems and TCP/UDP socket communication are required, as well as a message relay node that serves as a bridge between two different networks. In this case, SWEs send and receive their messages using the message relay node that is equipped with the RF modem of its own. The communication visibility in this type of connection is then partially determined using software models, and it is partially limited by the signal attenuation between the RF modems used for the actual message exchange.

## 4. Requirements for Communication Architecture

A communication subsystem in MR simulations needs to provide the following features to enable interaction between all the entities in an MR simulation and sustain the high fidelity of testing and verification.

- **Addressing**—Both SWEs and HWEs need to use unique addresses in a common address space to distinguish message receivers. In addition, messages need to have the option of being addressed selectively (not broadcast) so that (i) information is not given to entities that should not have it (e.g., when not covered by their sensors), (ii) the processing power of entities that are not receivers of the message is spared, and (iii), in some cases, wireless medium bandwidth is saved (e.g., when multiple wireless channels are used for communication or a directional information transfer methods are employed).
- **Transparent routing**—The system must provide the transparent routing of messages between SWEs and HWEs.
- **Identical message management**—Messages of HWEs and SWEs need to be managed similarly. Using different mechanisms for processing of HW and SW messages can bias tests regarding measurements of communication throughput, topology management, and team coordination.
- **Visibility model**—The system must be able to model communication visibility among all entities in the simulation (either based on their physical reachability or based on a signal propagation model) to provide high-fidelity simulations.
- **Network partitions**—In the case of two or more vehicles being out of communication range of the others and/or the simulation platform (i.e., GCS), the system should ensure that communication inside these newly created network partitions remain untouched.
- **Sharing single address**—When a communication bridge/relay between the networks of HWEs and SWEs is used, such entities need to be able to handle addressing using a single address of the bridge. It is necessary to determine the physical destination address of the messages that need to be relayed to other network types.

These features are important for the correct function of the MR simulation framework, high fidelity validation, and testing, and it is mandatory to compose them within a single architecture of an MR communication system, which is proposed in the following section.

## 5. The Proposed Architecture Design

**Figure 2:**
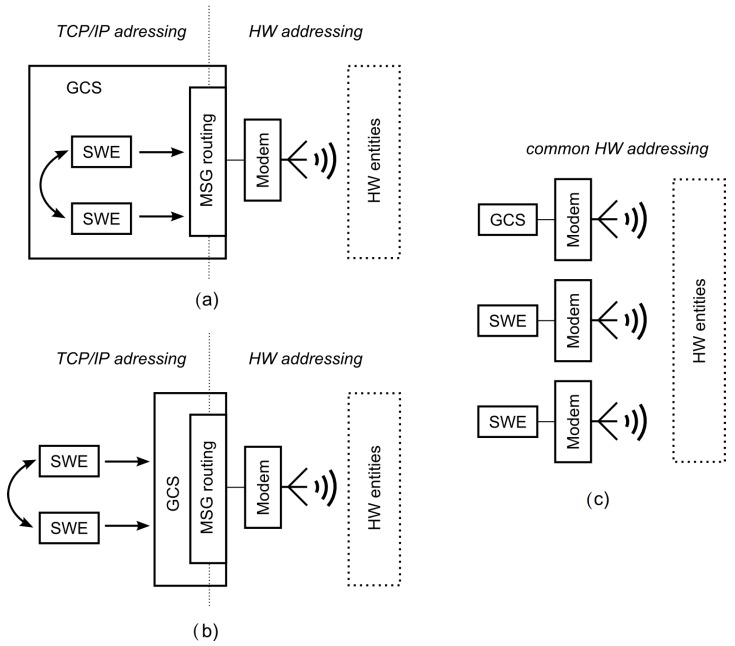
Various communication system architectures—(**a**) virtual entities within a ground control station (GCS); (**b**) virtual entities apart from the GCS that use it for message relay; and (**c**) separated modems for all entities.

**Figure 3:**
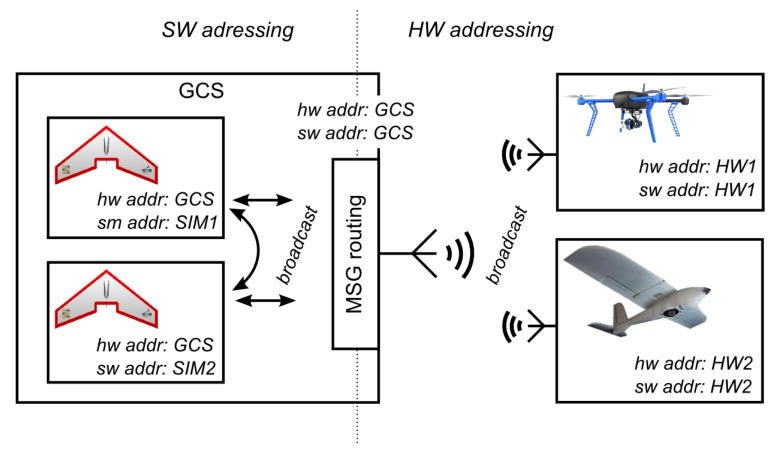
Dual addressing in MR communication system and distribution of *advertisment* messages.

Several topologies can be identified to fit the requirements of the communication system for the MR simulation that is defined in the previous section, and the most relevant topologies are depicted in Figure 2. A possible suitable topology uses two different networks (the network of HWEs and network of SWEs) with one bridge to relay the messages between them (labeled as *MSG routing* in Figure 2 and Figure 3). This bridge is represented by the simulation platform, i.e., which is most commonly the GCS. SWEs can be modeled either centrally within the GCS machine (as shown in Figure 2a), or individually in a separate machine that is connected to the GCS via TCP/UDP sockets to distribute the computational load on the simulation platform (as shown in Figure 2b). These topologies require only one communication modem to provide the simulation of all the SWEs; thus, they provide cheaper and less error-prone testbeds for high fidelity system validation. On the other hand, they require an addressing mechanism for routing in both the networks mentioned in the previous section. Further, they have to address the accuracy-based aspects of the communication model discussed in Section 6.

The second most related communication system topology uses a separate modem for each entity in the simulation (both SWEs and HWEs), so it creates one common network (as depicted in Figure 2c) with no need for any message routing and special addressing. Using RF modems for all entities also deals with the effect of using a single relay point on the bandwidth of the HWE–SWE connection discussed in Section 6.2. On the other hand, this topology requires an additional modem for every SWE in the system, so it decreases the scalability of the simulations and increases the cost. Modems are also susceptible to hardware-related errors, so this topology can be considered more error-prone compared to the previous case. Moreover, this topology breaks the concept of the purely virtual entities since the SWEs, in this case, could be considered as entities with the hardware-deployed communication layer. For these reasons, only the topologies with two communication networks are considered hereafter.

### 5.1. Addressing

Each entity needs to have two parts of its address to send messages between two different networks. The first part specifies the HW network address, i.e., the address of the physical RF modem at which it can be reached, and the second (SW) part specifies the entity’s address within the simulation and has to be unique for each entity. Information about these addresses needs to be propagated to all entities in both networks. A service similar to the IPv6 Neighbor Discovery protocol, as described in [41] can be used for that (alternatively, if the available bandwidth is an issue and multiple channels are available, a method similar to the cluster-based beaconing process [44] can be utilized). The router advertisement service has been adopted in the proposed architecture. This service has been modified to help with maintaining the reachability information of other active neighbors and the discovery of new entities in the environment. The entities periodically broadcast packets (*advertisements*) containing their ID and both their HW and SW address parts. After reception of such an advertisement, the entities update the list of their neighbors.

The message broadcast is the dissemination of a message to all reachable neighbors, both HWEs and SWEs. In the MR system, this means the usage of a standard broadcast method at the sender’s network (UDP broadcast in the SW network and RF transmission broadcast in the HW network) followed by a re-broadcast by the message relay node to the other networks upon message reception at the level of the GCS.

Figure 3 shows the principle of dual addressing on an example of two HW UAVs and two virtual ones modeled within the GCS. Here, all the communication participants have a unique SW address, and the SWEs share the HW address of the GCS.

### 5.2. Routing

**Figure 4:**
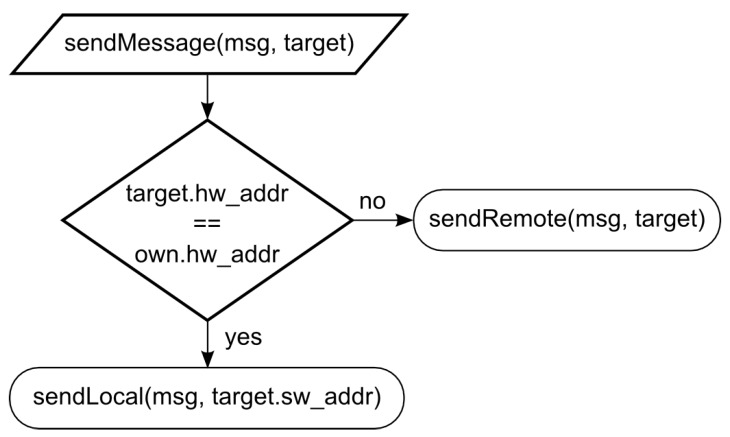
Routing principle in MR simulations.

Message routing used in the proposed architectures follows the flowchart depicted in Figure 4. If the target’s HW address part corresponds to the HW address of a transmitting entity, a mechanism of local message transport is utilized to deliver the message using the SW address part. We have used a combination of local IP addresses and TCP/UDP ports to represent the SW parts of the addresses and have used socket communication for local message transport. This applies to messages sent among SWEs. On the contrary, if the HW address parts of the target and the transmitting entity are different, a mechanism of remote message transport is employed to send the message to the appropriate HWE or GCS. When the HW part of the addresses is represented by the physical addresses of employed RF modems, the routing functionality provided by the modems can be utilized. If the utilization of specialized routing protocols is required, these protocols should be implemented outside of this architecture at higher control layers of the entities. Similar procedures are executed at the GCS during the message reception to provide the necessary message routing.

When using such a system, SWEs need to know where to send packets for the HWEs, since they do not have direct access to the GCS’s modem. The following two methods are proposed to provide this information. Both methods can be used for routing messages from SWEs to HWEs via the GCS. Each of the methods has its advantages and disadvantages that are detailed below.

- **Advertisement altering**—When re-broadcasting advertisements to the SWEs, the GCS changes both address parts of the advertising HWE to SW and HW addresses of this GCS while keeping a list that links the HWE IDs with their real addresses. Then, SWEs keep pairs of the HWEs’ IDs linked to the address of the GCS; thus, it sends the message directly to the GCS whenever an SWE has to send a message to an HWE. The main advantage of this approach is that no fixed gateway address needs to be specified for the SW nodes. On the other hand, altering every advertisement message is inefficient in scenarios with many HWEs.
- **Fixed gateway**—In this method, SWEs have a predefined gateway for HW addresses that they cannot connect to directly. In this case, the gateway address is the HW address of the GCS. This approach needs the gateway HW address to be defined and known by all SWEs before the start of the simulation, which may be limiting in some scenarios. However, apart from that, no further actions are needed for the correct message routing. Due to its simplicity and efficiency, this particular method with the fixed gateway for all SWEs is the selected approach adopted in the proposed architecture.

The routing methods mentioned above allow for the correct operation of the system even in cases when the network is partitioned into several groups, which is one of the requirements of the communication system of the MR simulations specified in Section 4. When two or more HWEs are out of the communication range of the others and/or the simulation platform (GCS), they can still communicate with each other, independently of the platform.

### 5.3. Visibility Model

**Figure 5:**
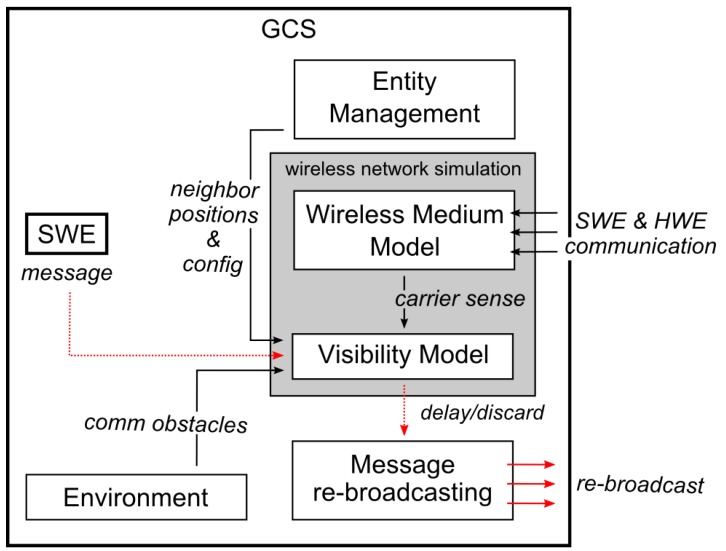
Visibility model for SWEs within the simulation platform (GCS) showing procesing of a message from one SWE.

The communication system in the MR simulation should provide the SWEs with a model of the wireless medium and simulate the neighbor communication visibility to provide a high fidelity simulation even on the message transport layer. Here, we propose employing a wireless network simulation in the communication subsystem of the MR simulation, as shown in Figure 5. In the proposed architecture, the simulation engine can be configured to intercept messages from SWEs and delay them when the model of the wireless medium signals is currently occupied. The messages are sent only to a subset of the receivers if they are not reachable at the moment; otherwise, the message is discarded completely, see Section 5.3.1 for further details. The evaluation of the delay and reachability of the message receivers is based on the presence of environmental obstacles, the positions of entities, their real or virtual communication equipment, the used MAC protocol, and a model of the actual utilization of wireless medium and RF signal propagation. An existing third-party wireless network simulator, e.g., OMNet++ [45] and GloMoSim [46], can be employed in the simulation for such a task, or a proprietary network simulation layer can be employed. A scheme of the proposed visibility modeling architecture within the simulation platform (GCS) of the MR simulation is depicted in Figure 5.

A short artificial delay is added to the transmission because the messages, together with an evaluation of the transmission delay and visibility of the entities, are sent to the wireless network simulator. This delay can be lowered by the utilization of the simulation architecture with the SWEs located within the simulation platform (see Figure 2a) since the message exchange among the platform and SWEs is minimized in such a case.

Even though SWEs transmit their messages inside the SW network, thanks to the simulation platform, these messages are re-broadcast within the real wireless medium. This way, a controlled RF signal interference is “injected” into the wireless channel, so there is no need to model the medium for the HWEs. These modeling properties are experimentally verified in Section 7.

#### 5.3.1. The Principle of Network Simulation Inside MR Simulations

**Figure 6:**
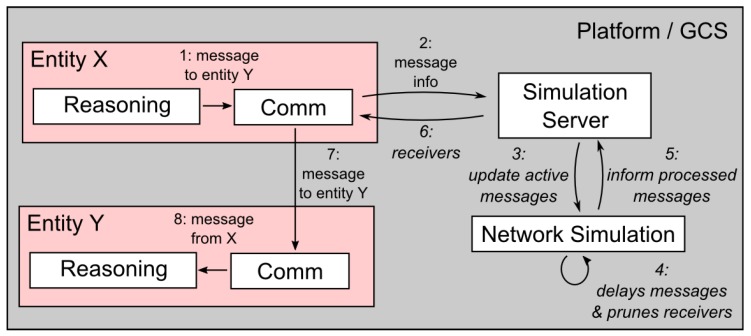
Principle of the deployment of the network simulation into MR simulations.

The principle of the deployment of network modeling features into the MR simulation is depicted in Figure 6. The figure shows the steps necessary to model the message transmission and reception in a situation when entity X sends a message to entity Y.

The steps are as follows.

1. The message is intercepted on the level of entity X’s *Comm layer*.
2. The digest of the message (sender, receiver(s), size, and transmission power) is sent to the *Simulation server*.
3. The *Simulation server* updates its network simulation model with this message digest.
4. The network simulation model decides (based on the information about the sender, receivers, obstacles, the actual wireless medium occupation, and the MAC protocol used) how much the message should be delayed and which receivers are reachable.
5. When the message is ready to be sent to their receivers, the network simulation model informs the *Simulation server*.
6. The *Comm layer* of entity X is informed that the message is ready to be sent.
7. The message is passed to the *Comm layer* of entity Y.
8. The message is received, and its content can be processed.

Using the architecture mentioned above provides the MR simulation testbeds with the capability of network simulation and entities that can move in real time according to their mission, operator commands, or, eventually, incentives from the environment. The network simulator reacts to the entities’ movement, and entities can change their plans according to the state of the network. Thus, no predefined traffic mobility models that cannot react to the changes caused by the entities’ interaction or operator commands are required. It should be noted that the movement of the communication nodes and their functionality, e.g., in the process of topology management, are to be specified at higher control levels that are not addressed by the proposed architecture.

## 6. Addressing the Precision Aspects of the Proposed Architecture

The proposed architecture enables validation process of complex multi-vehicle systems through high-fidelity testing in MR simulations. It also provides functionality of precise modeling of wireless channel utilization by (i) employing networking simulation for SWEs and (ii) injecting an RF signal into the wireless channel, as described in Section 5.3. Even though the process of injecting the RF signal into the wireless channel is necessary for the high-fidelity modeling of the medium utilization, it also produces undesirable side effects caused by the fact that multiple SWEs use a single static modem for the communication with HWEs. It produces interference that is inconsistent with the reality and could lower the precision of the simulations. These simulation aspects need to be addressed, and we propose the following solutions.

### 6.1. HWE–SWE Communication Reachability

**Figure 7:**
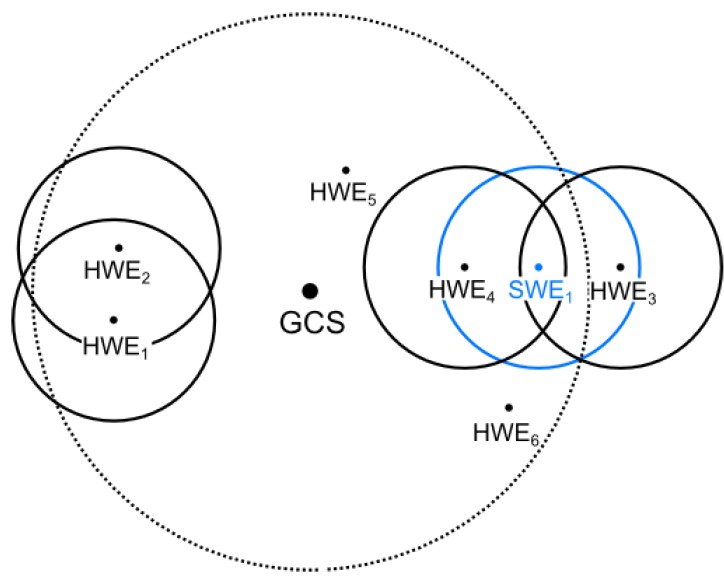
Illustration of configurations that could cause imprecise modeling of HWE–SWE reachability. Circles represent the communication ranges of individual entities and of the simulation platform (embodied by the GCS).

Messages exchanged between the SWEs and HWEs need to be routed via the modem of the simulation platform. Thus, the RF signal characteristics of the message transmission between HWEs and SWEs are given by the signal characteristics between the HWE’s modem and the modem of the simulation platform, and do not reflect the positions of the HWE and SWE in the simulation. This can result in situations where an HWE–SWE pair can be close to each other in the simulation, but the entities would be mutually unreachable for message transmission in reality since the HWE is too far away from the simulation platform. Similarly, an HWE–SWE pair could be far from each other in the simulation; however, if the HWE is close to the simulation platform, its messages can be delivered. The main issues in the communication reachability of HWEs and SWEs that can occur are illustrated in Figure 7 and are as follows.

1. Nodes SWE${}_{1}$ and HWE${}_{1}$ **should not be** mutually visible, but **they are**.
2. Nodes SWE${}_{1}$ and HWE${}_{3}$ **should be** mutually visible, but **they are not**.
3. Communication between nodes SWE${}_{1}$ and HWE${}_{4}$ **should not** interfere with the communication between nodes HWE${}_{1}$ and HWE${}_{2}$, **but it does**.

We propose the following precautions that should be applied to address the identified issues and to achieve the high-fidelity simulation of the communications.

1. A proper model of the RF signal propagation should be used for the message transmission between the HWE–SWE pairs to limit the message receivers to those that can be reached. This can be done during the message re-transmission issued by the simulation platform. The particular mechanism of the wireless network simulation that can be used to address this aspect is described in Section 5.3.
2. Range testing of the simulation scenarios needs to be limited to the area that is reachable for the signal of the particular simulation platform’s modem. The modem has to be equipped with a high gain antenna to provide as large a testing area as possible. This way, no HWE can get outside of the range of the simulation platform and thus out of the range of any SWE (unless it is caused by the signal propagation model mentioned in the previous case).
3. Undesired signal interference caused by the simulation platform affects all communications in the reachable area of the platform’s modem. It cannot be avoided; only its effects are limited. Two particular precautions can be taken. First, the transmit power of the platform’s modem can be adjusted for every transmission and set to the minimal required power for a successful message reception; therefore, it does not influence nodes further away from the platform. For example, if the power is set to transmit a message to HWE${}_{4}$ in Figure 7, HWE${}_{6}$ would not be affected; however, HWE${}_{5}$ would still be. Second, the simulation platform’s modem can be equipped with multiple directional antennas and a mechanism for using only selected ones for message transmissions based on the knowledge of positions of the target entities. However, the results of these two particular precautions have not been reported in the herein presented evaluation results. They are the subject of our future work.

### 6.2. Bandwidth of a Single Relay Point

Available transmission data rates of all entities are influenced by the use of a single message relay point, the simulation platform. The MR simulation is thus prone to three bandwidth modeling issues. The first issue is caused by the fact that SWEs do not use the RF modems directly. Thus, there are fewer modems than there are entities, so there is less RF noise than there would be in real scenarios. The second issue is caused by the communication among SWEs that is not affected by the limitations of the RF medium and modems. Hence, SWEs could theoretically send data at much higher data rates than entities in real scenarios. The third issue can arise from the fact that the platform’s modem needs to transmit data for all SWEs, and this may require higher data rates than the modem is physically capable of achieving.

The following proposed precautions should be taken to address the simulation precision issues caused by the single relay point in MR simulations.

- Transmission delays caused by the medium access and back-off mechanisms need to be emulated. This is done by utilizing the medium access control (MAC) layer for SWEs as shown in Figure 5. Moreover, the arrival of HW messages at the simulation platform needs to be monitored and integrated into the MAC layer model to limit the available bandwidth of the SWEs. Obviously, this behavior is dependent on the MAC protocol used, whether they are contention-based, contention-free [22], or hybrid MAC protocols [23] suggested for VANETs. Section 5.3 describes the mechanism of the wireless network simulation that can be used to address this simulation aspect.
- The broadcasting nature of the wireless channel traffic needs to be emulated, and the bandwidth of HWEs needs to be limited. In the proposed architecture, this is achieved by injecting controlled RF signal interference. Specifically, all message transmissions between SWEs need to be re-broadcasted over the simulation platform’s modems as stated in Section 5.3. This, however, results in the issues of undesired interferences discussed in Section 6.1.
- Transmission data rates of all SWEs must be limited not to collectively request to transmit more data per second than the modem used by the simulation platform is physically capable of transmitting. This is achieved by using a high data-rate modem for the simulation platform, limiting the number of simultaneous large data transmissions, or, if the previous cases are not applicable, by limiting the number of SWEs in the simulation. Another option to prevent large simultaneous data-rate requests is to ensure the distribution of SWE transmissions over time to comply with modem limitations. However, this last approach has not yet been implemented and is a subject of the future work.

A comparison of the reached data rates in the case of hardware deployed entities and in the case of entities in an MR simulation that employs the proposed architecture and precautions is the main subject of the experiments presented in the following section.

## 7. Experiments

The main motivation of the work presented herein is to support the development of complex UAVs via communication simulation, employing the concept of MR simulations. Our goal is to provide the same communication characteristics of systems using only HWEs in systems using SWEs. Therefore, we designed the experiments to validate the proposed communication architecture and to show that the communication characteristics of a testbed configuration with solely HWEs present can be substituted with the characteristics of MR testbeds where some of the HWEs are modeled using SWEs. The experiments measuring the data rates were chosen since they well illustrate the behavior of the system in various settings. This behavior is expected to be similar when other communication characteristics are measured.

### 7.1. Testbed Configurations

**Figure 8:**
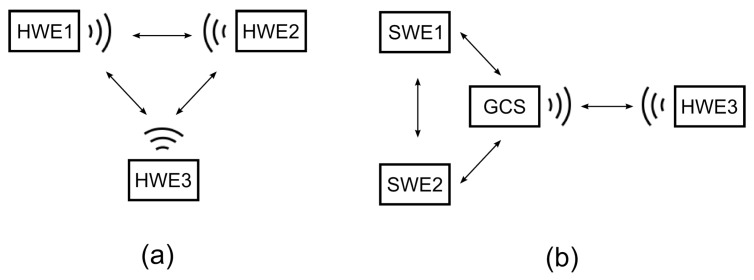
Two experimental scenarios—(**a**) the three-HWE (HW) scenario with three hardware entities and (**b**) the MR scenario where one hardware and two simulated entities communicate via GCS relay.

Two testbed configurations are considered in the presented results for the proposed experimental setting: one with three HWEs, which is hereafter referred to as the HW scenario, and one with the simulation platform (represented by the GCS) to communicate with one HWE, and two remaining HWEs modeled in the MR simulation. The latter configuration is hereafter referred to as the MR scenario. These two configurations are schematically depicted in Figure 8. The MR simulation used the architecture with SWEs modeled within the simulation platform according to Figure 2a.

The main evaluation indicator of the communication characteristics is measured as the number of bytes per second successfully transferred between the three entities. All entities have been static and positioned at the same distances from each other. During the experiments, all of the entities broadcasted packets of 1 kB at a rate of 15 kBps using the UDP protocol, and successfully received data was measured every second for each entity.

Three Microhard nVIP2400 modems with the TX power set to 14 dBm were used as the communication hardware. One of the modems (in particular the one utilized by HWE${}_{3}$) was equipped with an antenna that was superior to the others (8 dBi vs. 2 dBi). Thus, in a case of simultaneous communication of all entities, it was capable of transmitting approx. 12 kBps through the wireless medium, while the other two modems (used by HWE${}_{1}$, HWE${}_{2}$, and by the GCS in the second testing configuration) were only capable of successfully transmitting approx. 9 kBps in the HW scenario.

### 7.2. Results

**Figure 9:**
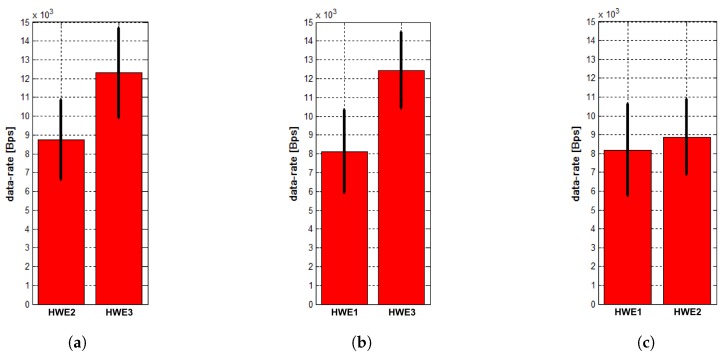
Mean values and standard deviation of data rates of transmissions from neighbors measured at entities (**a**) HWE${}_{1}$; (**b**) HWE${}_{2}$; and (**c**) HWE${}_{3}$ in the HW scenario.

The mean values and standard deviations of the measured data rates in the HW scenario are shown in Figure 9. The results indicate the above-proclaimed transmission data rates of the HWEs.

**Figure 10:**
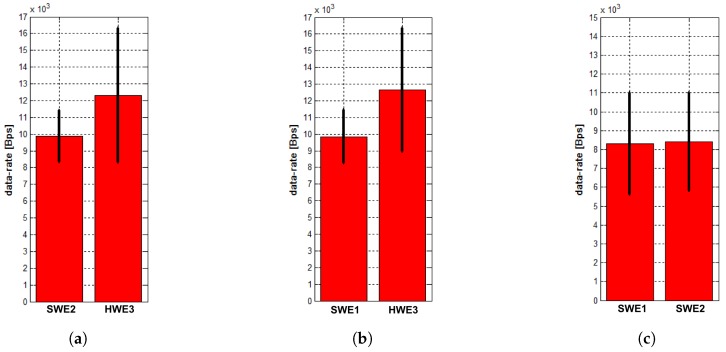
Mean values and standard deviation of data rates of transmissions from neighbors measured at entities (**a**) SWE${}_{1}$; (**b**) SWE${}_{2}$; and (**c**) HWE${}_{3}$ in the MR scenario using the high-fidelity MR architecture.

In the MR scenario, the GCS uses the modem of the HWE${}_{1}$ from the previous case. The measured average data rates and standard deviations are shown in Figure 10. The characteristics are almost similar to those from the first experiment. The data rates measured between the two SWEs are higher with a lower variance, which may be caused by a low precision of the employed wireless network model and by the fact that the environmental background noise has not been taken into account during the simulation. On the other hand, the variances of the real traffic (data from HWE${}_{3}$ as shown in Figure 10a,b, and data from SWE${}_{1}$ and SWE${}_{2}$ shown in Figure 10c) are higher. This is most likely caused by the MAC in situation when the GCS’s modem tries to send twice as much data compared to the modems in the HW scenario. The similarity in the characteristics of the SWE transmissions arises from the fact that the same modem is used for the message exchange; thus, the system operates in similar conditions during the transmission. It is obvious that, in this configuration, the GCS’s modem needs to transmit twice as much data compared to the first experiment since it is now transmitting data for both SWEs. However, thanks to the lower bandwidth occupancy caused by one missing modem, and because the model is physically capable of transmitting this amount of data, the packets are transmitted with almost no change in bandwidth in comparison with the first experiment. The results of this experiments demonstrate that both configurations can be used without a significant difference nor with any effect on simulation accuracy.

**Figure 11:**
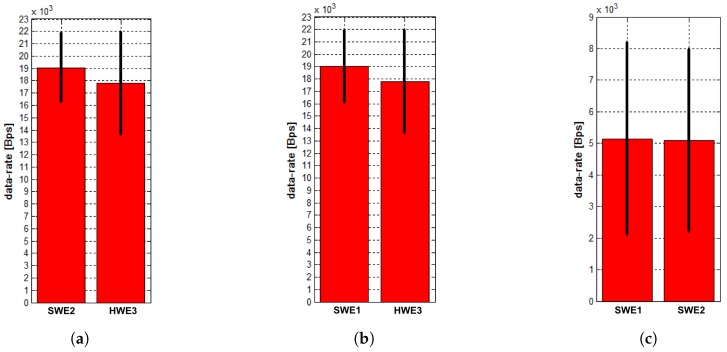
Mean values and standard deviation of data rates of transmissions from neighbors measured at entities (**a**) SWE${}_{1}$; (**b**) SWE${}_{2}$; and (**c**) HWE${}_{3}$ in the MR scenario without the use of the high-fidelity MR architecture when data is sent at a rate of 20 kBps.

Besides the first comparison, we also performed MR experiments with the testbed configuration similar to the MR scenario, but without any wireless network modeling, and the UDP packets were sent at a rate of 20 kBps to see how the system would behave in situations where the entities are trying to send more data than the real RF modems can transmit without any regulatory mechanism. The achieved results are depicted in Figure 11.

According to Figure 11c, the results indicate what happens when the modem tries to send more data of which it is physically capable. There is a significant drop in transmitted data rates and a significant increase in their variance. The Microhard modems have their MAC mechanisms, but they are not satisfactory in this case, and many packets are lost. SWEs transmit much more data between each other than with the HWE (as can be seen in Figure 11a,b), and the simulation of the communication cannot be used for high-fidelity validation of the communication system.

### 7.3. Example of Simulation Performance on Physical Layer

**Figure 12:**
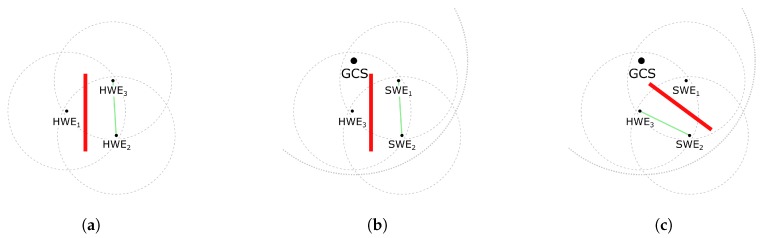
Scenarios showing the system behavior during message exchange under the presence of a communication obstacle (red). In all cases, each entity tries to send a message to the remaining two. The messages are exchanged only between entities that have mutual obstacle free connectivity (green lines). The rest of the messages are dropped either directly by the effect of the obstacle in the HW scenario or by the network simulation within the GCS in the MR scenario.

The performance of the MR simulation in the PHY layer is demonstrated in Figure 12. The message receivers are pruned when three nodes send messages to each other and when an obstacle that blocks the transmission is placed between them. In all cases where SWEs are present, the wireless network simulation block would (as shown in Figure 5 and Figure 6) intercept all messages from and for SWEs and prune their receivers according to their visibility from senders. Note that, for correct operation of this functionality, the GCS needs to be aware of the obstacles and have connectivity with the HWEs, as specified in Section 6.1.

## 8. Deployment Examples

The herein described communication architecture has been proposed to enable development and final evaluation with a practical deployment of several multi-vehicle systems (mostly aircraft, i.e., UASs) that used the MR simulations for incremental development and validation. In this section, we describe three particular representative applications to support the benefits of the proposed communication architecture.

**Figure 13:**
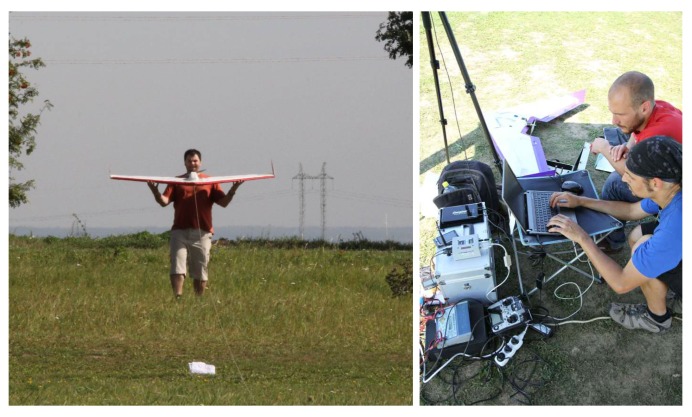
Flight tests of the multi-UAV system.

One of the use cases of the proposed architecture occurred while an unmanned system that provides command and control capabilities over a heterogeneous team of autonomous unmanned aircrafts was being developed [16,17]. These UAVs are capable of mutual communication and cooperation while they perform complex tactical missions, such as perimeter protection, surveillance, and target tracking. Our system was finally deployed on multiple delta-wing UAVs, see Figure 13.

Apart from the obvious features, such as the interaction among all of the entities in the simulation, the utilization of the proposed communication architecture provides additional benefits. For example, we were able to measure the requirements of the communication devices used by the UAVs in the MR simulation before purchasing the necessary hardware communication equipment. Further, the features of the message routing and visibility modeling helped us to study the effects of the UAV communication unreachability on the team management in the simulation, which increased the scalability of the whole system. Moreover, using this architecture allowed us to seamlessly move from the pure virtual simulation to the full HW deployment and back according to actual development needs.

**Figure 14:**
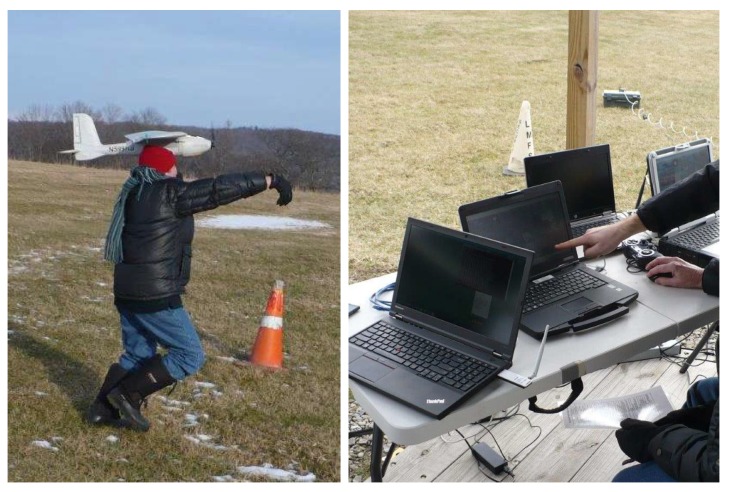
Flight tests with Desert Hawks III.

The second application was to a system for verifying communication-based cooperative collision avoidance methods among multiple fixed wing UAVs. In this case, the objective was to develop a complete testing environment by incrementally deploying the system on two Lockheed Martin’s Desert Hawk III aircrafts and training the system operators. An example of the Desert Hawk III launch and field test settings is shown in Figure 14. Here, the communication architecture allowed us to design simulation scenarios with communication requirements similar to those in the case of hardware deployment, and thus allowed us to safely study the effects of communication delays on the developed collision avoidance mechanisms. Further, the proposed architecture allowed for the seamless deployment of the system from the virtual environment to the hardware assets. The operator training process was thus smooth and safe.

**Figure 15:**
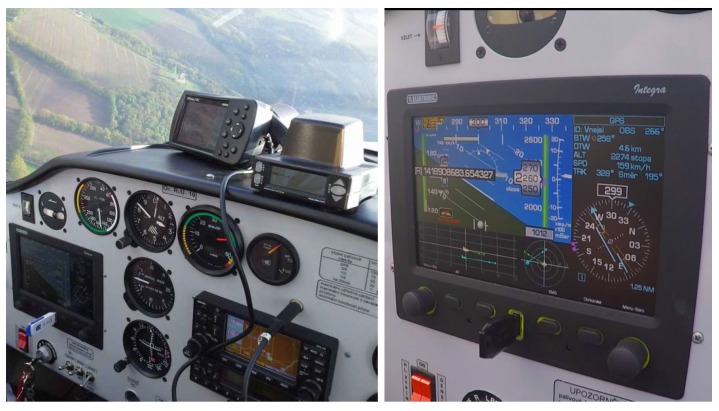
Aircraft pilot panel with the integrated advisory system—built-in EFIS Integra, Zaon PCAS with GPS on the flight panel and the rest of the system integrated under the panel.

Finally, the third application was to a system for providing increased flight safety in the domain of light-sport aircrafts, where a combination of existing sensors, negotiation among vehicles, cooperative, and non-cooperative methods of collision avoidance and trajectory planning algorithms are utilized to recommend the best collision-free trajectories for pilots or autopilots of such aircrafts [15]. The final deployment of the advisory system on a Tecnam P-2002 Sierra aircraft is shown in Figure 15. In this case, the communication architecture was used to allow for an MR simulation with both the real and virtual aircrafts, and this was very helpful during the development of such a complex system. Further, the architecture’s advertisement system was utilized so that new aircraft in the neighbourhood could be discovered, which is applicable in real scenarios where the aircraft cannot benefit from a GCS for communication mediation.

Even though the use cases hitherto mentioned are from the area of aeronautics, the proposed architecture can be utilized for other vehicular systems that directly communicate with other vehicles (vehicle-to-vehicle, V2V). Only the RF signal propagation models need to be updated (e.g., to reflect problems of RF signal propagation close to the ground), the movement dynamics models of SWEs need to be changed (e.g., to allow car-like movement), and the HWEs need to be replaced with real vehicles used in the target systems (e.g., to use real car-like vehicles instead of UAVs).

The architecture can also be theoretically used for the simulation of systems that utilize communication with the infrastructure (vehicle-to-infrastructure, V2I), where the infrastructure elements would be represented as separate SW or HW entities. However, such a generalization needs further verification and has to deal with additional precision aspects.

## 9. Conclusions

The MR simulations are beneficial for the development of multi-vehicular systems, as has been shown in the past by several works including our previous effort on developing UAVs. It is mostly beneficial for the validation of the correct function of complex vehicular systems using high-fidelity testbeds. In this work, we have presented requirements and proposed a design for a communication architecture of MR simulations that allows for the co-existence and interaction of virtual and physical entities within a single multi-vehicle system. Suitable architecture topologies have been proposed, and their advantages and disadvantages for various system configurations have been discussed. Methods of addressing and routing in two different communication networks used in the MR simulations have been presented. The precision aspects of the wireless medium utilization have been addressed, and the method of integration of wireless network simulation capabilities have been described.

The proposed resulting system can be utilized as a high-fidelity network simulator for vehicular systems with implicit mobility models that are given by real trajectories of the vehicles. In particular, this means that the mobility of entities does not have to be modeled in advance, and entities move in real time according to their mission, operator commands, or, eventually, incentives from the environment. Moreover, the MR simulations provide the benefits of high-fidelity field experiments, but at a lower cost and with fewer risks, since some of the entities in the system are virtualized. The herein proposed architecture has been already successfully used for the development and validation of several multi-aircraft systems. To the best of our knowledge, no similar architecture for MR simulations has been presented.

The experiments were designed to validate the proposed communication architecture and have shown that the communication characteristics of a testbed configuration with hardware entities can be substituted with the characteristics of MR testbeds where some of the entities are modeled. Thus, both configurations can be used without a significant difference and effect on simulation accuracy.

In future work, we would like to extend the approaches discussed in Section 6. In particular, we aim to consider the adaptation of the dynamic transmit power of the simulation platform’s modem and exploit the usage of multiple directional antennas and distribution of large transmissions over time to prevent transmission requests from virtual entities that would collectively exceed the available data rates of the platform’s modem.

## Figures and Tables

**Table 1:** Types of communication connection in MR simulation systems.

Type	Tools	Visibility	Method
HWE–HWE	modems	physical	direct (modems)
SWE–SWE	sockets	SW model	direct (sockets)
HWE–SWE	combined	SW models (limited by physical vis)	message relay

## Abbreviations

The following abbreviations are used in this manuscript:

FANET	flying ad-hoc network
GCS	ground control station
HW	hardware
HWE	entity embodied in the physical world
MAC	medium access control
MANET	mobile ad-hoc network
MR	mixed-reality
RF	radio-frequency
SW	software
SWE	virtual entity
UAV	unmanned aerial vehicle
UAS	unmanned aerial system
V2I	vehicle-to-infrastructure
V2V	vehicle-to-vehicle
VANET	vehicle ad-hoc network
